# Rapid screening of TCR-pMHC interactions by the YAMTAD system

**DOI:** 10.1038/s41421-022-00386-2

**Published:** 2022-04-05

**Authors:** Lihui Wang, Xun Lan

**Affiliations:** 1 Department of Basic Medical Science, School of Medicine, Tsinghua University, Beijing, China; 2 Tsinghua-Peking Joint Center for Life Sciences, Tsinghua University, Beijing, China; 3 MOE Key Laboratory of Bioinformatics, Tsinghua University, Beijing, China

**Keywords:** Biological techniques, Immunology

## Abstract

Personalized immunotherapy, such as cancer vaccine and TCR-T methods, demands rapid screening of TCR-pMHC interactions. While several screening approaches have been developed, their throughput is limited. Here, the Yeast Agglutination Mediated TCR antigen Discovery system (YAMTAD) was designed and demonstrated to allow fast and unbiased library-on-library screening of TCR-pMHC interactions. Our proof-of-principle study achieved high sensitivity and specificity in identifying antigens for a given TCR and identifying TCRs recognizing a given pMHC for modest library sizes. Finally, the enrichment of high-affinity TCR-pMHC interactions by YAMTAD in library-on-library screening was demonstrated. Given the high throughput (10^6^–10^8^ × 10^6^–10^8^ in theory) and simplicity (identifying TCR-pMHC interactions without purification of TCR and pMHC) of YAMTAD, this study provides a rapid but effective platform for TCR-pMHC interaction screening, with valuable applications in future personalized immunotherapy.

## Introduction

Adoptive cell therapy (ACT) is a promising strategy in the treatment of advanced cancers and viral infections^1–3^. Current ACT treatment relies on the isolation of tumor-reactive T cells from tumor-infiltrating lymphocytes (TILs)^4,5^, limiting its application in “cold” tumors that have less lymphocyte infiltration. Genetically modified T cells expressing antigen-specific T-cell receptors (TCRs) can be applied to a more extensive scope of patients^6–9^, including patients with “cold” tumors, but the generation of high-affinity TCRs is currently difficult and limits their applications.

One way to generate high-affinity TCRs is phage display- or yeast display-mediated TCR evolution^10–13^. Yeast display of a CDR3α-mutated library has been successfully applied to identify high-affinity TCRs that recognize pMHCs with a lower input of antigens^14–21^. The risk of cross-reactivity and off-target recognition increases along with affinity^22,23^. Therefore, evaluating the cross-reactivity of high-affinity TCRs is essential to the safety of TCR-T therapy. Additionally, both cancer cells and viruses evolve quickly to escape their elimination by high-affinity T cells. Rapid screening of multiple immunogenic antigens and associated TCRs can reduce the chance that tumors or viruses develop resistance to T cell therapy.

Three high-throughput methods for T cell antigen discovery were developed: pMHC tetramer-based screening, cell-based screening, and yeast display-based screening. The tetramer method uses pMHC multimers that are DNA-barcoded to detect TCR specificities. However, pMHC tetramers require prior knowledge about antigenic targets. In addition, such methods face challenges including low-throughput peptide synthesis, low efficiency in pMHC multimer production, instability of pMHC multimers, a limited library size, and biases in PCR amplification^24^.

For cell-based assays such as trygocytosis^25,26^, T-scan^27^ helps screen the TCR-pMHC interaction by evaluating the function of T cells after coincubation of T cells with pMHC-expressing cells. These methods can screen approximately 10^4^–10^5^ epitopes simultaneously. Although no previous knowledge of the antigen is required, experimental deconvolution is required to match these T cells with cognate antigen-presenting cells (APCs).

Previous studies have also reported a technique using yeast display of pHLA libraries to discover TCR ligands. This method requires no prior knowledge of those antigens and can be scalable to 10^6^–10^8^ epitopes. However, the TCR of interest must be expressed in the TCR tetramer form, which is difficult to purify^28–30^.

Here, we developed YAMTAD by combining yeast display and yeast mating to enable library-on-library screening of the TCR-pMHC interaction with high affinity. Integrating the TCR and pMHC cassettes into the yeast genome provides constitutive expression and achieves a near-perfect surface display level. We designed a yeast T cell coculture system to quickly confirm pMHC function without using TCR tetramers. Our proof-of-principle study demonstrates that the YAMTAD system can be used for screening experimentally confirmed TCR and pMHC interactions (868 TCR- SL9 HLA-A*02:01) in peptide libraries and in TCR libraries with high sensitivity and specificity. Finally, we show that YAMTAD can identify high-affinity TCR-pMHC interactions in unbiased library-on-library screening.

## Results

### Design of YAMTAD, a high-throughput system to screen TCR-pMHC interactions

**Fig. 1:**
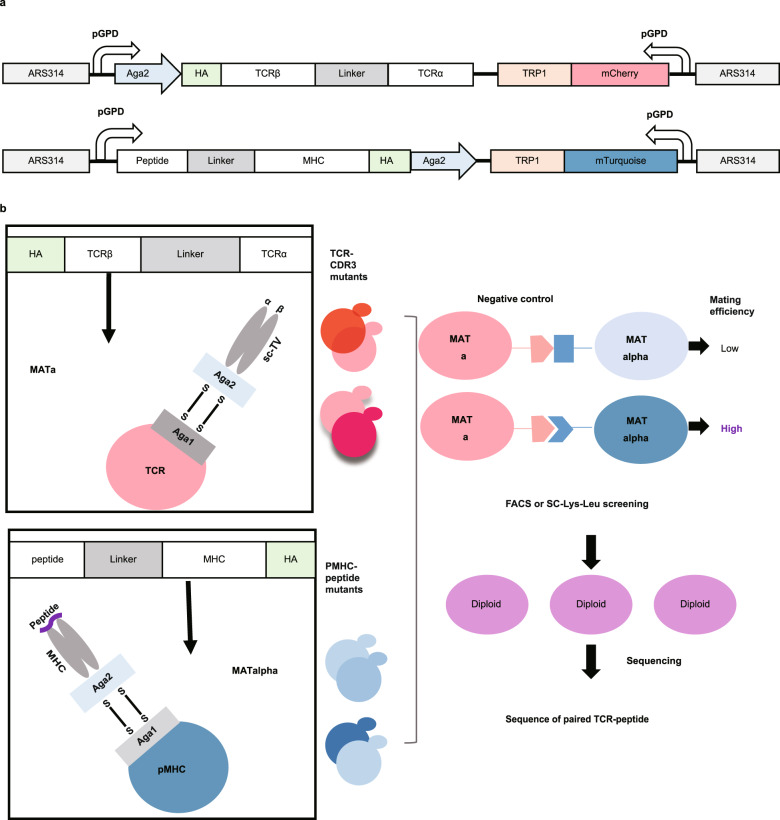
Schematic overview of the YAMTAD system. **a** Schematic diagram of the strategy used for constructing the TCR and pMHC cassettes. The TCR cassette was constructed by connecting the variable domains of the TCR with a flexible linker. The pMHC cassette features an N-terminal peptide library linked to wild-type β-2-microglobulin (β2M), followed by the HLA heavy chain. TCR and pMHC cassettes are tethered to an epitope tag and Aga2p driven by the constitutive GPD promoter (pGPD). TCR and pMHC cassettes and mating-type-specific fluorescence reporters (mCherry and mTurquoise) were integrated into the ARS314 site of MATa or MATalpha yeast, respectively, using TRP1 as the selection marker. **b** Schematic overview of the YAMTAD system. TCR and pMHC cassettes were displayed on MATa and MATalpha yeast cell surfaces by binding to the Aga1 protein. MATa and MATalpha have complementary leucine and lysine auxotrophic markers for diploid selection. Mating is induced when a TCR displayed on MATa yeast recognizes cognate pMHC displayed on MATalpha yeast. The diploids expressing the double fluorescence can be sorted by FACS or SC-Lys-Leu screening for identification of the peptide and TCR sequences in them by next-generation sequencing.

Yeast display can reach a library size of 10^8^ and thus is sufficient to cover the entire sequence space of TCR and pMHC^29^. The yeast mating system was previously applied to probe protein-protein interactions (PPIs)^31^ and thus is ideal for TCR-pMHC interaction identification. The yeast agglutination-mediated TCR antigen discovery system (YAMTAD) was designed herein by combining these features to screen TCR-pMHC interactions in a high-throughput way (Fig. 1).

First, the TCR cassette was constructed in the reported single-chain TCR (scTv) format^32,33^ in which the variable domains of the TCR were connected by a flexible linker (Vβ-linker-Vα, Fig. 1a, top). pMHC features an N-terminal peptide library linked to wild-type β-2-microglobulin (β2M), followed by the HLA heavy chain^29^ (Fig. 1a, bottom). TCR and pMHC cassettes, driven by the constitutive GPD promoter (pGPD), were tethered to an epitope tag and Aga2p. TCR-CDR3 mutants and pMHC-peptide mutants can be generated based on these cassettes.

Second, these two constructs were integrated into the genome of yeasts with opposite mating types separately, together with a mating-type-specific fluorescent reporter, using TRP1 as the selection marker (Fig. 1b). MATa yeast expresses TCR-CDR3 mutants and mCherry and has a leucine auxotrophic marker. MATalpha yeast expresses pMHC-peptide mutants and mTurquoise and has a lysine auxotrophic marker. The complementary lysine and leucine auxotrophic marker can be further used for diploid selection. TCR and pMHC cassettes were displayed on yeast by binding to the constitutively expressed Aga1 protein. Once the two haploid cells were cocultured in complete medium-YPD for 22 h, a higher mating efficiency between yeasts occurred when a TCR expressed on MATa yeast recognized the cognate pMHC expressed on MATalpha yeast compared with the negative control.

Finally, we selected diploids with double fluorescence by FACS or in SC-Lys-Leu medium and identified the sequences of peptides and TCRs in them by next-generation sequencing. The identified candidates were further validated.

### Genome integrated cassettes are efficiently displayed

We used two previously known TCR-pMHC pairs to determine the efficiency of the YAMTAD system. One is the 868 TCR, which recognizes a 9-amino-acid sequence (SL9, SLYNTVATL) derived from the HIV-1 Gag antigen bound to HLA-A*02:01^34^. The other is the A6 TCR, which recognizes a 9-amino-acid sequence (TAX, LLFGYPVYV) derived from the HTLV-1 antigen bound to HLA-A*02:01^34^.

**Fig. 2:**
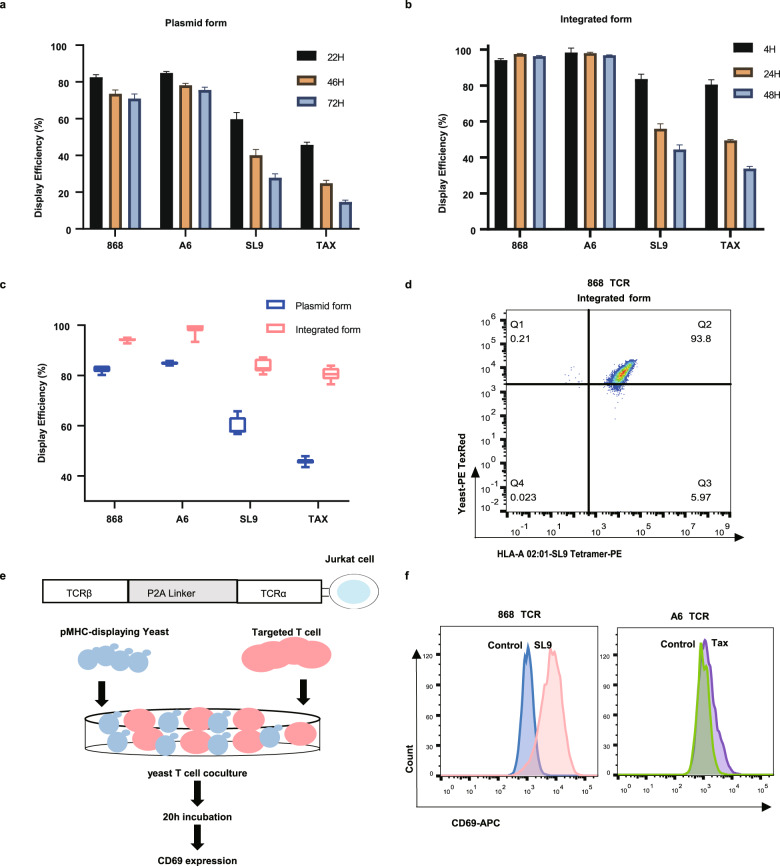
Constitutively expressed TCR and pMHC achieved near 100% display efficiency. **a**, **b** The display efficiencies (mean ± sd) of TCRs (868 or A6) and pMHCs (SL9-HLA-A*02:01 or TAX-HLA-A*02:01) expressed in plasmid form (**a**) or integrated form (**b**). The display efficiency was measured by FACS after staining the cells with HA-FTIC antibody at the indicated hours. Each experiment was performed with 2 technical repeats and was independently duplicated 3 times. The mean of the 6 duplicates is shown. **c** Comparison of the highest display efficiencies achieved among the different time points for the plasmid and the integrated forms. **d** The 868 TCR expressed in the integrated form recognized the SL9-HLA-A*02:01 tetramer. The SL9- HLA-A*02:01 tetramer was labeled with PE, and mCherry in MATa yeast was detected using PE-TexRed. **e** A schematic diagram of the yeast T cell co-culture system. The cognate T cells were constructed by stable integration of a TCR β and a TCR α gene, which were linked by the P2A linker. The pMHC-displaying yeast was the same as shown in Fig. 1a. pMHC-displaying yeasts were cocultured with cognate T cells for 20 h, followed by FACS to detect CD69 expression in T cells using an APC-conjugated CD69 antibody. **f** The 868 T cells and A6 T cells can be activated by cognate pMHC-displaying yeasts. Histograms showed staining by the APC-conjugated CD69 antibody. Representative data from one out of three biological replicates are shown.

Consistent with previous studies, when the Aga2p-fused TCR^34,35^ and pMHC^29^ were separately ligated into the pCTon2 plasmid and displayed on the EBY100 yeast strains, TCR was detected on 70–80% of the yeast cell surfaces after induction for 22 h, while the highest display efficiency for pMHC was only 40–60% (Fig. 2a, c). Prolonged induction failed to improve the display efficiencies but decreased them, which was probably due to the loss of plasmids during cultivation (Fig. 2a, c). Thus, the TCR and pMHC cassettes were integrated into the ARS314 sites of ySYNAGa and ySYNAGalpha^31^ to test whether a higher display efficiency could be achieved (Fig. 1a). The display efficiency of TCR was significantly increased to near 100% and remained steady with prolonged culture time (Fig. 2b, c). The display efficiency of pMHC was also increased by ~20%, but the highest efficiency appeared only after induction for 4 h and subsequently decreased (Fig. 2b, c). The gating strategy of flow cytometry and original data are shown in Supplementary Fig. S1. Another TCR-pMHC pair, i.e., 2 C TCR-QL9 (QLSPFPFDL) bound to H–2Ld^16^ (Supplementary Fig. S2a), was also efficiently displayed using the integrated form, claiming its universality.

Together, genome integration and constitutive expression of the TCR and pMHC significantly improved the display efficiency.

### Yeast-displayed TCR and pMHC are functional

To confirm whether the displayed TCRs were functional, we used the SL9-HLA-A*02:01 tetramer (labeled by PE) to stain the displayed 868 TCR. We found that 868 TCR expressed in the integrated form efficiently recognized the SL9-HLA-A*02:01 tetramer (Fig. 2d). Using the anti-2C-TCR antibody, we also confirmed the proper folding of the 2 C TCR (Supplementary Fig. S2b).

Moreover, we designed a yeast T cell coculture system to confirm the function of yeast-displayed pMHC (Fig. 2e). After a 20 h incubation of pMHC-displaying yeast together with the cognate T cells, the expression of CD69, a T cell early activation marker, was detected by FACS. We found that yeast displaying SL9-HLA-A*02:01 and TAX-HLA-A*02:01 could activate cognate T cells (Fig. 2f). Moreover, the proportion of activated T cells was associated with the TCR-pMHC interaction affinity. The low-affinity TCR, A6 TCR, was activated at a lower level than the high-affinity 868 TCR (Fig. 2f). The gating strategy for flow cytometry is shown in Supplementary Fig. S3a. These results highlighted that the yeast T cell coculture system assay enables quick functional verification of pMHC displayed on the yeast surface without purifying the TCR tetramer, which is expensive and time-consuming.

Yeast with SL9-HLA-A*02:01 expressed in the plasmid form activated 33% of the 868 T cells. In contrast, the integrated constitutively expressed form activated 75.6% of the 868 T cells (Supplementary Fig. S3b). In addition, the highest T cell activation level was achieved when the effector cell (E)-T cell/target cell (T)-pMHC-displaying yeast cell ratio was 1:3 (Supplementary Fig. S3c). These results indicated that the display efficiency of pMHC on yeast was a factor impacting the activation of T cells. Therefore, increasing the display efficiency is essential for the YAMTAD system to achieve high sensitivity in screening TCR-pMHC interactions, the affinity of which is lower than other types of protein-protein interactions.

**Fig. 3:**
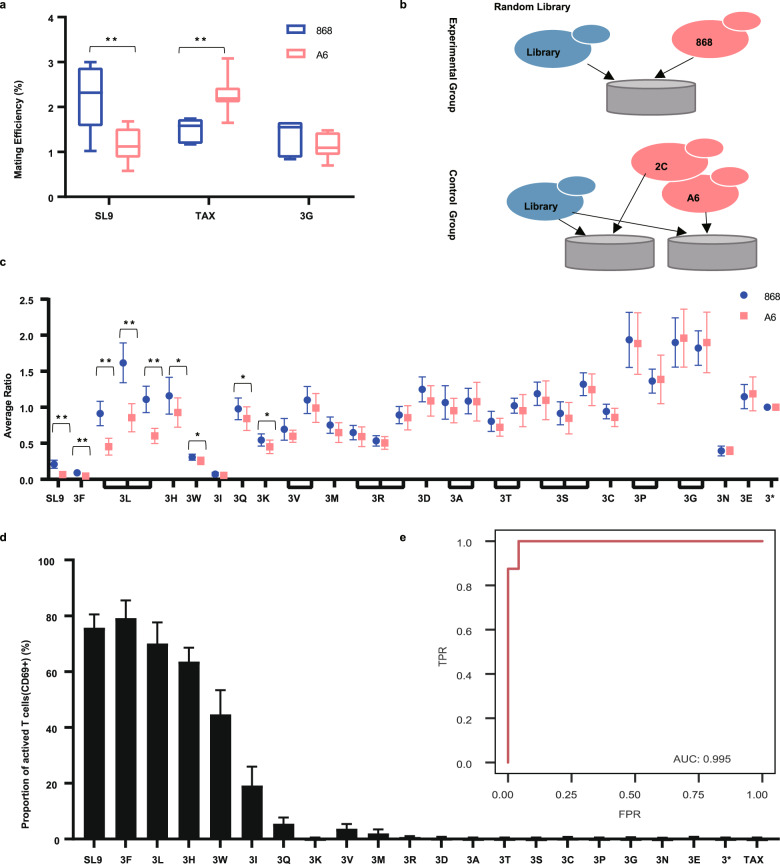
The YAMTAD system can be used to screen cognate peptides for a given TCR. **a** The mating efficiency (mean ± sd) between the cognate TCR-pMHC pairs was significantly higher than that of the negative control pairs. The mating efficiency was calculated by the percentage of diploids detected by FACS. Seven biological repeats were measured. **b** A schematic diagram of the strategy used for screening cognate peptides for a given TCR in a randomly generated peptide library. The random peptide library was mated with MATa yeasts expressing the 868 TCR as the experimental group and with MATa yeasts expressing the A6 or 2 C TCR as the control group. **c** Ratios (mean ± sd) of the indicated SL9 mutants to the 3* peptide in diploid yeasts induced in YAMTAD screening. Each experiment was performed with 4 technical repeats and was independently duplicated 3 times. The mean of 12 duplicates is shown. **d** Proportion (mean ± sd) of 868 T cells activated by various SL9 mutants in the yeast T cell co-culture system. The Y-axis represents the proportion of activated T cells (CD69+) after coculturing with the indicated yeast strains. **e** The ROC curve depicting the FPR (false-positive rate, 1-specificity) and TPR (true positive rate, sensitivity) for library screening of TCR-pMHC interactions using the YAMTAD system. We defined a peptide as a cognate peptide if it could activate more than 10% of the T cells on average in the yeast T cell coculture assay. Unpaired two-tailed Student’s t-test was used for statistical analysis in **a**, **c**. **Indicates a two-tailed *P* value < 0.01. *Indicates a two-tailed *P* value < 0.05.

Whether TCRs and pMHCs displayed on the surface of yeast can mimic the specific TCR-pMHC interaction is unknown. By optimizing the initial OD, mating conditions, and the volume of the medium to increase the adhesion time (see Materials and Methods), we showed that the agglutination-dependent mating efficiency between cognate pairs was significantly higher than that of the negative control pairs (Fig. 3a and Supplementary Fig. S4a, b). Interestingly, the YAMTAD results showed that 868 TCR failed to recognize the 3 G mutant carrying a mutation of the third amino acid of SL9 to glycine (Fig. 3a). This finding was further confirmed by the yeast T cell coculture system (Fig. 3d, Supplementary Fig. S4c, d).

To summarize, we successfully displayed functional TCR and pMHC on the surface of yeast at high efficiency, which allowed the YAMTAD system to detect TCR-pMHC interactions.

### The YAMTAD system can be used to screen cognate peptides for a given TCR

To further test whether the YAMTAD system can be used for screening cognate peptides for a given TCR in a randomly generated peptide library, we constructed a random library of various mutants at the third amino acid of the SL9 peptide and transformed the library into the ySYNAGalpha strain (Supplementary Fig. S5a, b). The pMHC peptide mutants were mated with MATa yeast expressing 868 TCR or A6/2 C TCR (Fig. 3b). Using the 3* mutant as an internal control for normalization, which could not be recognized by any TCR due to the introduction of a stop codon into the third amino acid of SL9, SL9,3 F, 3 L,3H, 3 W, 3Q, and 3 K were significantly enriched (Fig. 3c, Supplementary Table S1, and Materials and Methods), suggesting that they had higher affinity to 868 TCR. The raw data before normalization are shown in Supplementary Fig. S5c and Table S1.

To confirm whether the peptide could be recognized by the 868 TCR, the interaction was verified using the established yeast T cell coculture system. We found that the enriched SL9 and its variants, 3 F, 3 L, 3H, 3 W, and 3Q, could activate the 868 TCR, while 3 K could not activate the 868 TCR (Fig. 3d). Among these mutants, SL9, 3 F, 3 L, 3H, and 3 W achieved higher T cell activation, which was also associated with the higher affinity determined by tetramer staining (Supplementary Fig. S6a, b), a method used widely for affinity assessment^36^. The difference between the activation value and the KD value may be due to the impact of the surface display efficiency on the activation of T cells in the yeast T cell co-culture system (Supplementary Fig. S6c). The receiver operating characteristic (ROC) curve was generated to depict the relationship between false-positive rate (FPR) and true positive rate (TPR) for library screening of TCR-pMHC interactions using the YAMTAD system. Here we defined a peptide as a cognate peptide if it can activate more than 10% of the T cells on average in the yeast T cell co-culture assay. The ROC curve suggested that YAMTAD can achieve satisfactory specificity and sensitivity in the screening of high-affinity TCR-pMHC interaction (Fig. 3e).

However, the 3I mutant activated a higher proportion of T cells but was not detected in the system, possibly due to its low presence in the random library (Supplementary Fig. S5b). Although SL9 and the 3 F mutant also had low proportions, their affinity to the cognate TCR was much higher. The 3 M and 3 V mutants activated a much lower proportion of cognate T cells, and the YAMTAD system failed to enrich these peptides. These results suggested that the sensitivity of the YAMTAD system to detect a TCR-pMHC interaction was influenced by both the affinity of the pair and the proportion of the peptide in the random library.

To summarize, our results demonstrated that the YAMTAD system can be used to screen cognate peptides for the 868 TCR with high specificity (85.8%) and sensitivity (66.7%) in an unequally distributed random library, which can be applied for evaluation of the cross-reactivity of a TCR.

### The YAMTAD system can be used to screen cognate TCRs for a given peptide

**Fig. 4:**
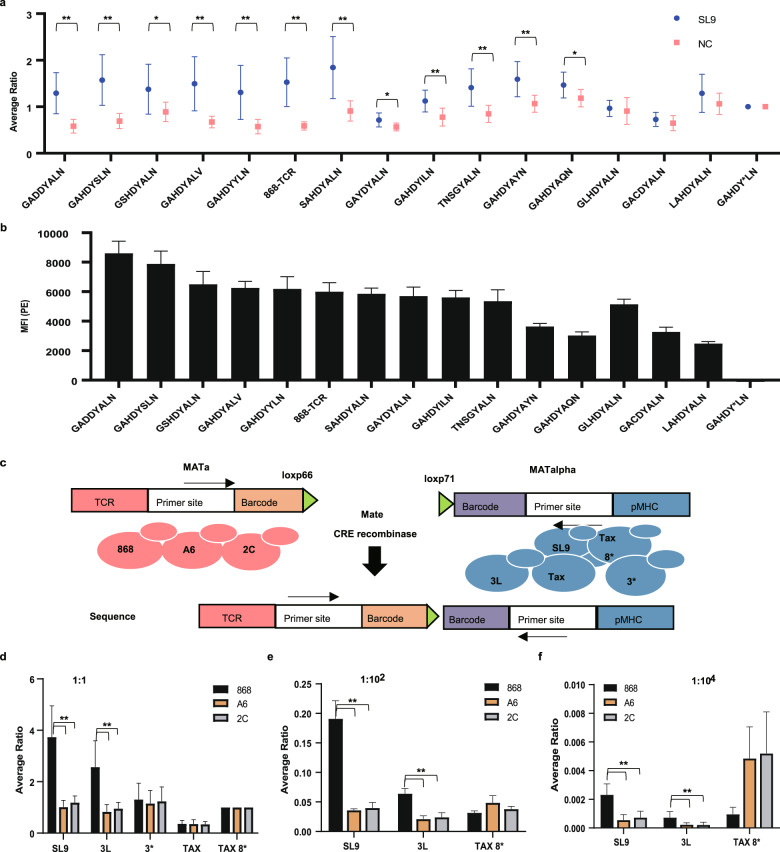
YAMTAD system for cognate TCR screening and library-on-library screening. **a** Ratios (mean ± sd) of the 868 TCR mutants to the GAHDY*LN TCR in diploid yeasts induced in the YAMTAD screening. The NC peptides include TAX and 3*. Each experiment was performed with 4 technical repeats and was independently duplicated 2 times. The mean of 8 duplicates is shown. **b** The median MFI (mean ± sd) of the indicated 868 TCR mutants stained with PE fluorescently labeled SL9-HLA-A*02:01 tetramer. **c** A schematic diagram of the barcoding strategy used in library-on-library screening. A unique barcode was added to each TCR or pMHC strain. Expression of Cre recombinase induces recombination between the loxp 66 and loxp 71 sites, resulting in a juxtaposition of the MATa and MATalpha barcodes on the same chromosome. The two barcodes could be amplified by specific primers and subsequently sequenced simultaneously. **d** Ratios (mean ± sd) of diploids induced by the indicated peptide-TCR pairs to diploids induced by the negative control, the peptide-TAX 8* -TCR, in the YAMTAD library-on-library screening. The five peptides used for screening were mixed equally. Each experiment was performed with 4 technical repeats and was independently duplicated 3 times. The mean of 12 duplicates is shown. **e** Ratios (mean ± sd) of diploids induced by the indicated peptide-TCR pairs to diploids induced by the negative control, the peptide-3* -TCR, in the YAMTAD library-on-library screening. The SL9, 3 L, and TAX 8* peptides were mixed with the negative yeasts-3* and TAX at a 1:10^2^ ratio. Each experiment was performed with at least 3 technical repeats and was independently duplicated twice. The mean of 7 replicates is shown. **f** Ratios (mean ± sd) of diploids induced by the indicated peptide-TCR pairs to diploids induced by the negative control, the peptide-3* -TCR, in the YAMTAD library-on-library screening. The SL9, 3 L, and TAX 8* peptides were mixed with the negative yeast- 3* and TAX at a 1:10^4^ ratio. Each experiment was performed with at least 3 technical repeats and was independently duplicated twice. The mean of 7 replicates is shown. Unpaired two-tailed Student’s t-test was used for statistical analysis in **a**, **d**, **e**, **f**. ** indicates a two-tailed *P* value < 0.01. * indicates a two-tailed *P* value < 0.05.

Next, we wondered whether the system could be used for high-affinity TCR mutant screening. Fifteen VαCDR3 868 mutants were constructed and mated with MATalpha yeast expressing SL9-HLA-A*02:01 or TAX-HLA*02:01/SL9 3*-HLA-A*02:01. TAX-HLA-A*02:01 and SL9 3*-HLA-A*02:01 were used as negative controls (NCs) for screening to exclude noise to obtain truly interacting TCR-pMHCs (Fig. 4a). As shown before, we used the VαCDR3 mutants with a stop codon (GAHDY*LN) to perform the normalization. By using the fold change of SL9/NC > 1 and P-value < 0.05 as cutoffs, the positive control-868 TCR and almost all the VαCDR3 mutants except GLHDYALN, GACDYALN, and LAHDYALN (Fig. 4a and Supplementary Table S2) were enriched. The raw data before normalization are shown in Supplementary Fig. S7 and Table S2.

Then, by using the SL9 tetramer to confirm the interaction, we found that all of these enriched mutants could be stained with the SL9 tetramer, which indicated that the mutants could recognize the SL9 peptide (Fig. 4b). However, unenriched mutants could also be stained by the SL9 tetramer but seemed to have a relatively lower median fluorescence intensity (MFI) (Fig. 4b), indicating a relatively lower affinity of these three mutants. Therefore, we concluded that the YAMTAD system has the potential to screen cognate TCRs for the SL9 peptide with high specificity, but the sensitivity (80%) needs to be improved.

### The YAMTAD system can be expanded to library-on-library screening

To further test whether YAMTAD could achieve unbiased library-on-library screening, we mixed the TCRs (868, A6, and 2 C) and pMHC peptide mutants (SL9, 3 L, 3*, TAX, and TAX 8*) together at a 1:1 ratio. All yeast strains displaying various TCRs and pMHCs were labeled with random DNA barcodes (Supplementary Fig. S8a). For each of the 868, A6, and 2 C TCRs, two strains with distinct barcodes were generated to exclude possible variations between barcodes. The matching between the sequences of TCRs/pMHCs and the sequences of their labeling barcode was determined by targeted amplification in the haploid strains followed by Sanger sequencing (see Materials and Methods). The barcode sequence of the barcode-labeled yeasts is shown in Supplementary Fig. S8a. The MATa strain expressed Cre recombinase, and the MATalpha strain expressed ZEV4, a β-estradiol inducible transcription factor for activating CRE recombinase expression^31^. After mating, the expression of Cre recombinase was induced with β-estradiol in the diploid to generate recombination between the loxp66 locus in the MATa strain and the loxp71 locus in MATalpha, which resulted in the conjugation of the two barcodes from two mating strains at the juxtaposition on the same chromosome. Using specific primers, the two barcodes were subsequently amplified and sequenced simultaneously (Fig. 4c)

Next, we calculated the average proportions of each peptide mutant that appeared with the 868/A6/2 C TCR and then normalized the numbers based on the proportion of the TAX 8* peptide, introducing the stop codon into the eighth amino acid of TAX in each group. We found that 3 L/SL9 was significantly enriched in the 868 TCR group but not in the A6 or 2 C TCR group (Fig. 4d and Supplementary Table S3). The raw data before normalization are shown in Supplementary Fig. S8b and Table S3. Our results suggested that the high-affinity TCR-pMHC interactions with a KD value of 3.81 nM (868 TCR-SL9) and 132 nM (868 TCR-3L) can be enriched in library-on-library screening.

To further test the sensitivity and specificity of the YAMTAD system in large library-on-library screenings, TAX 8*, SL9, and 3 L were respectively mixed with the other negative yeasts, including, TAX and 3*, at 1:10^2^ and 1:10^4^ ratios to generate synthetic libraries. We then performed YAMTAD screening in a scaled-up mating volume (see Materials and Methods). Next, we calculated the average proportions of each peptide mutant that appeared together with the 868/A6/2 C TCR and normalized the numbers based on the proportion of the 3* peptide, which serves as an internal negative control by introducing a stop codon at the third amino acid of SL9. We found that 3 L and SL9 were significantly enriched in the 868 TCR group but not in the A6 or 2 C TCR group at the 1:10^2^ (Fig. 4e) and 1:10^4^ (Fig. 4f) dilution levels, suggesting that the YAMTAD system can potentially be used to identify TCR-pMHC interactions in large libraries. The raw data are shown in Supplementary Table S3.

## Discussion

The YAMTAD system detects TCR–pMHC interactions by combining yeast display and engineered yeast mating. Our proof-of-principle study demonstrates that YAMTAD can systematically evaluate the cross-reactivity of TCR and screen the cognate TCR and pMHC in a library-on-library way without purification of any protein.

Yeast display is a powerful technique for T cell antigen discovery. However, previous studies have shown that when either TCR or pMHC is displayed on yeast, the affinity of the TCR-pMHC interaction was lower than that determined by using surface plasmon resonance with their soluble form^17^. Most studies have used the inducible promoter-pGAL to induce expression of the displayed protein; however, the display efficiency is low using this construct. In the YAMTAD system, we constitutively expressed TCR and pMHC and integrated them into the yeast genome, leading to a near-perfect display efficiency of TCR and pMHC. Combining a better display level and optimized mating conditions, the YAMTAD system was applied for effective TCR-pMHC interaction screening.

We showed that YAMTAD enabled rapid identification of peptide or TCR mutants using one-pot screening of designed libraries rather than individual testing of protein pairs. When screening in the random library, we used the golden gate cloning method so that the pMHC yeast library could be constructed within 2 weeks without synthesizing peptides or engineering cell lines, which is costly and time-consuming. Moreover, a library incorporating random variants of an epitope can be used to interrogate TCR cross-reactivity and to identify altered peptide ligands or heteroclitic peptides. In addition, we established a yeast T cell coculture system in which pMHC-displayed yeast directly activated cognate T cells. It is a convenient method to simultaneously test the display efficiency and the immunogenic function of pMHC displayed on the surface of yeasts.

By using the Cre-loxP system, we can induce chromosomal translocation in diploid cells, resulting in a cis arrangement of the barcodes of the TCR and pMHC, which can be sequenced simultaneously. As a proof of principle, we showed that YAMTAD could identify TCR and pMHC interactions in unbiased library-on-library screening with high specificity. The sizes of the yeast libraries of TCR and pMHC can be scaled up to 10^6^–10^8^ in theory, which is considerably higher than tetramer-based (10^3^) and cell-based approaches (10^4^–10^5^).

Currently, the sensitivity of the YAMTAD system for finding interactions with low affinity is suboptimal. Three factors may have played a role herein: a. reduced mating efficiency due to low affinity between the TCR and pMHC; b. low stability of the displayed proteins on the yeast surface; and c. certain mutants that may be underrepresented in the library. Further optimization in the mating condition is warranted to increase the system’s sensitivity.

## Materials and methods

### DNA construction

The sequences of 868 TCR, A6 TCR^34^, 2 C TCR^16^, and peptide/HLA-A*0201^29^ were synthesized by the Beijing Genomics Institute (BGI).

For expression in plasmid form, all TCR and pMHC genes were excised from the synthesized plasmid using NheI-XhoI and ligated into the NheI-XhoI-digested pCTon2 plasmid. The pCTon2 plasmid was a gift from Dr. Linqi Zhang at Tsinghua University, Beijing, China.

The plasmids used for the integrated constitutive expression of the TCR or pMHC were based on the ysyna_Dest or ysynalpha_Dest plasmid, which were donated by Dr. Eric Klavins from the Electrical Engineering Department at the University of Washington, United States. The plasmid contained the ARS314 homologous site for integration, the TRP1 gene as the selection marker, and the mCherry or mTurquoise gene as the reporter gene. All TCR and pMHC genes were constructed using the Gibson Assembly and confirmed by Sanger sequencing. In short, the ysynalpha_Dest and ysyna Dest plasmids were linearized by NheI and XhoI. The TCR and pMHC were amplified by PCR to introduce the overlap region for the linearized plasmid. The following cloning procedure was performed according to the 2× seamless Clonekit (Beyotime Biotechnology) protocol.

All plasmids used in this study were listed in Supplementary Table S4. Sequences for all cloning primers, fragments, and plasmids are available upon request.

### Library construction

To construct the random peptide library, the ysynalpha_Dest plasmid was reconstructed. These BsaI sites were removed from the ysynalpha-pMHC plasmid, and the peptide region was replaced with two BsaI sites. The plasmid can be used as the backbone for peptide library construction. The random peptide library was constructed by Golden Gate assembly as previously reported^37^. In short, primers for DNA-encoded peptide libraries were synthesized with NNK codons at chosen codon sets and flanked by two BsaI sites. Then, the random peptide library was amplified with high-fidelity 2X Vazyme Mix (Vazyme) using random primers. The PCR conditions and program were performed following the Vazyme mix protocol: 95 °C for 3 min, 10 cycles of 95 °C for 15 s, 55 °C for 15 s, 72 °C for 20 s, followed by 72 °C for 5 min.

The ‘one-pot’ assembly reaction was performed using the accepting vector and the purified PCR with the following composition (for the 50 µl mixture): 5 µl 10 × T4 ligase buffer, 5 µl 10× bovine serum albumin (BSA), PCR product 262.8 ng, 50 ng plasmid, 1.25 µl BSAI (NEB), 0.5 µl T4 ligase (Thermo Scientific) and an appropriate volume of ddH_2_O. The reaction conditions were 37 °C for 3 h, 50 °C for 20 min, 80 °C for 15 min and maintenance at 15 °C.

After accumulating sufficient ligation products, several rounds of electroporation into *E. coli* Top10 were performed following ethanol precipitation of the DNA to obtain the peptide bacterial library. Twenty clones were picked to perform Sanger sequencing to calculate the efficiency of the correct insertion. The diversity of the bacterial library was calculated by multiplying the number of clones after limiting dilutions and the efficiency of the insertion. All clones were pooled, and maxi plasmid purification (TIANGEN) was performed to obtain the plasmids used for random peptide library construction.

For library-on-library screening, we constructed different strains with specific barcodes. The plasmid containing the TCR or pMHC with a unique barcode was generated by the Gibson Assembly. The αCDR3 TCR mutants were also generated by Gibson Assembly. In short, the barcode and CDR3 mutants were imported into the DNA using the primer and the NNK codon with the overlapped sequence for the linearized plasmid. All NNK primers’ sequences used can be found in Supplementary Table S6. The ligation conditions followed the 2× Seamless Clone kit (Beyotime Biotechnology) protocol. After Sanger sequencing, we matched the specific barcodes with the different strains.

### Yeast strain construction

All yeasts were constructed based on the parental strains MATa and MATalpha donated by Dr. Eric Klavins from the Electrical Engineering Department at the University of Washington, United States. These strains, as variants of the EBY100 strain, were constructed with mating, sporulation, tetrad dissection, and screening with selectable markers^31^. Finally, 868, A6, SL9, and TAX strains were constructed by chromosomal integration, consisting of digestion of the targeted plasmid with Pme1 and performance of standard lithium acetate transformation selection on the SC-TRP plate. The relevant strains expressed in plasmid form were constructed by standard lithium acetate transformation.

The random peptide library was constructed as previously described, which consisted of digesting the library plasmids with Pme1 and performing several rounds of lithium acetate transformation. We selected twenty clones to detect mCherry or mTurquoise expression to confirm the correct integrated efficiency using an LSRFortessa flow cytometer. The diversity of the library was calculated by multiplying the numbers of clones after limiting dilutions and the efficiency of the insertion. The pooled yeast library was mixed at 10× diversity of the individual length libraries and frozen at −80 °C in 15% glycerol. The library contained 10^4^ colonies, representing 1000× the theorized capacity. All these yeast strains used in this study were listed in Supplementary Table S5.

### Surface expression

To induce surface expression of the TCR or pMHC expressed in the plasmid form, we first cultured the strains in SDCAA (pH 4.5) medium to an OD_600_ = 2.0 − 4.0. Then, we shifted the strains to SGCAA medium starting from OD_600_ = 1 and cultured them at 20 °C for different times to induce protein expression. For integrated constitutive expression of the TCR or pMHC, we directly cultured these strains overnight and diluted them to OD_600_ = 0.1 for different times to induce protein surface display.

To measure the display efficiency for the TCR/pMHC or the function of the TCR, 0.1 OD of the induced yeast cells were washed with 1 mL PBS + 1% BSA and incubated in 30 μL PBS + 1% BSA media with FITC-anti-HA antibody (1:200, Invitrogen) or the SL9-HLA-A*0201 tetramer (1:200, MBL) for 30 min at room temperature, washed with 1 mL PBS + 1% BSA and read with the LSRFortessa flow cytometer. The data were analyzed using FlowJo software.

### Cell line construction

A total of 868 TCR and A6 TCR genes carrying human TCR constant regions were constructed in the format TCRβ-P2A-TCRα. The TCR was cloned into a lentiviral pHAGE-IRES-RFP vector (a gift from Dr. Xin Lin at Tsinghua University). L-293T cells, Jurkat cells with a native TCR deletion, were also a gift from Dr. Xin Lin at Tsinghua University.

Retroviruses encoding 868-TCR and A6-TCR were produced in L-293T cells by transient transfection of lentiviral-based vectors and their packaging vectors (psPAX2 and pMD2.G). At 48 h after transfection, we collected the virus supernatant and used it for infection after filtering through a 0.45-µm syringe filter.

Jurkat cells were spin-infected with viral supernatant at 1500 rpm at 32 °C for 90 min. On Day 3 postinfection, the transfected Jurkat cells were sorted using an Arial SORP flow cytometer and cultured for use in the subsequent coculture assay.

### Coculture assay

We mixed 1 × 10^5^ targeted T cells and appropriate yeast cells according to the E:T ratio in a 12-well plate and cultured them in RPMI 1640 medium (Gibco) supplemented with 10% (v/v) FBS at 37 °C and 5% CO_2_. After 20 h of coculturing, the cells were stained with anti-CD69-APC (1:200, BioLegend) and analyzed using an LSRFortessa flow cytometer.

### Growth curve

To measure the growth curve, overnight cultured yeast cells were diluted to an OD_600_ = 0.1 in a final volume of 10 ml using fresh YPD medium and cultured at 30 °C and 220 rpm. Each strain was measured in triplicate. At different time points, we measured the OD_600_ with a spectrophotometer (YouKe, Shanghai, China).

### Mating assays

Possibly due to the low-affinity nature of TCR-pMHC interactions^16^, agglutination-dependent mating was not detected between TCR- and pMHC-displaying yeasts using previously reported conditions^31^ (Supplementary Fig. S4a). We optimized the mating condition to successfully detect the TCR-pMHC interaction.

A single clone of MATa or MATalpha haploid yeast strain was cultured overnight. We diluted the strain to OD_600_ = 0.1 and cultured it in 2 ml YPD medium for 4 h to reach an OD_600_ = 0.4–0.55. Then, we mixed a(0.0125OD) and alpha (0.0375OD) in 1 ml medium in a Deep-well Multiwall plate with a pointed bottom and cultured the mixture at 30 °C, 220 rpm for 20 h. Since the affinity of A6-TAX was lower than that of 868-SL9, we adjusted the mating condition by mixing the haploid strains that grew to the late log phase.

After mating, we diluted 5 µl of the strain in 400 µl PBS + 1% BSA medium to perform LSRFortessa flow cytometer analysis. A standard yeast gate was applied to all cytometry data, and FlowJo was used for analysis and visualization. The population expressing double fluorescence was defined as diploid, and its percentage was regarded as the percentage of diploid. To confirm that the region was diploid, we sorted MATa, MATalpha, and diploid cells. After culturing, we extracted the genome of the strains and used MATa/alpha-specific primers to confirm the mate type^38^. The three primers were both added to perform the PCR.

In the random library screening, the diploid was selected using SC-Lys-Leu medium after 22 h of mating. In short, 500 µl of the strain was washed twice in 1 mL SC-Lys-Leu medium and then transferred to 25 mL SC-Lys-Leu medium for culturing for 24 h. For library-on-library mating, the mating medium was shifted to SC-Lys-Leu medium with 1 µM β‐estradiol (Sigma) for diploid selection and induction of Cre recombinase. After 24 h of growth, genomic DNA was extracted for NGS. For screening with the synthetic library at a 1:10^2^ ratio, we mixed the SL9 (0.000375OD), 3 L (0.000375OD) and TAX 8* (0.000375OD) with the negative yeasts-3* (0.01875OD) and TAX (0.01875OD). The synthetic library was then mixed with 868 TCR (0.004OD), A6 TCR (0.004OD) and 2 C TCR (0.004OD). To ensure enough yeasts for the screening, 3 mating wells were cultured for each repeat. After mating, 2 ml of yeast was washed twice in 1 mL SC-Lys-Leu medium and then transferred to 50 mL SC-Lys-Leu medium with 1 uM β‐estradiol (Sigma) for culturing for 24 h. For screening with the synthetic library at a 1:10^4^ ratio, the SL9 (3.75 × 10^−6^ OD), 3 L (3.75 × 10^−6^ OD) and TAX 8* (3.75 × 10^−6^ OD) with the negative yeasts-3* (0.01875OD) and TAX (0.01875OD) were mixed. The synthetic library was then mixed with 868 TCR (0.004OD), A6 TCR (0.004OD) and 2 C TCR (0.004OD). 43 wells were performed for each repeat. After mating, 25 ml of yeast was washed twice in 1 mL SC-Lys-Leu medium and then transferred to 500 mL SC-Lys-Leu medium with 1 uM β‐estradiol (Sigma) for culturing for 24 h.

### Genome extraction

All the collected yeast cells were washed once with sterile water and suspended in 300 μL breaking buffer (10 mM Tris-Cl, pH 8.0, 100 mM NaCl, 1 mM EDTA, pH 8.0, 2% (v/v) Triton X-100, 1% (w/v) SDS). Then, approximately 300 μL of 0.5 mm glass beads (Biospec, 11079105) was added, followed by the addition of 600 μL of phenol/chloroform/isoamyl alcohol (25:24:1). The cells were shaken on a mini-shaker (ALLSHENG, Hangzhou, China) for 3 h. After adding 300 μL sterile water, 550 μL of the top layer was collected by centrifugation at 14,680 rpm for 10 min, transferred into a new tube to precipitate the DNA by adding 1 ml 100% ethanol and stored at −20 °C for 30 min before centrifugation (13,000 rpm, 5 min at 4 °C). After washing the pellet once with 500 μL of 75% ethanol, the genomic DNA was dissolved in 200 µl of sterile water and stored at −20 °C. The volume of all reagents used for genome extraction was scaled up according to the amount of yeasts.

### Preparation for NGS

Two rounds of PCR were performed to amplify the peptide or the TCR CDR3 mutants from the genomic DNA and to add standard Illumina sequencing adaptors and the index. For the primary PCR, different primers were used for amplifying the peptide mutants, the TCR CDR3 mutants, and the specific barcode used for the library-on-library screening. An index barcode was added in the secondary PCR. All NGS primers were listed in Supplementary Table S6.

The first PCR was performed using KAPA HiFi HotStart ReadyMix (KAPA BIOSYSTEMS) and was run for 30 cycles. After gel purification of the PCR fragment using the Zymoclean Gel DNA Recovery Kit (Zymo), the concentration of DNA was quantified using a Qubit 3 Fluorimeter (Invitrogen). The second PCR included 500 ng DNA from the first round as a template and was run for 5 cycles. Then, we also purified the PCR fragments using the Zymoclean Gel DNA Recovery Kit (Zymo), quantified them with a Qubit 3 Fluorimeter, and sequenced them with a Nova Platform sequencer (GENEWIZ and Novogen).

### Sequence analysis

After DNA sequencing, we extracted the mutated sequence (27 bp) after obtaining a perfect match of GTTTTTCAATATTTTCTGTTATTGCTAGCGTTTTAGCA (38 bp). Similarly, the CDR3 mutants (24 bp) were extracted after obtaining the perfect match of GATTCTGCTACTTATTTGTGTGCTGTTAGA (30 bp). Then we summed the sequence reads for the mutants and calculated the individual proportions for each mutant. To obtain the normalized average ratio, the reads were divided by the reads of the negative control (TAX 8*, 3* or GAHDY*LN). After obtaining the average ratio, we compared the value in the experimental group with that in the control group and performed a *t*-test to assess the statistical significance.

To analyze the sequence data for the library-on-library screening, all the sequences were first filtered by quality. Then, the barcode sequences (15 bp for each end) were extracted after the conserved sequence ‘GAAAAGCGGC’ (forward) and the conserved sequence ‘CCAGGTATCG’ (reverse). The barcodes were later used to match the TCR and pMHC mutants. Sequences were then grouped based on TCR barcodes and used to calculate the individual proportion for each mutant. The same TCR strains with different barcodes were identified as the same group, and the average proportion for each pMHC mutant was calculated. To obtain the normalized average ratio, the reads were divided by reads of TAX 8*. After obtaining the average ratio, we compared the value in the experimental group with that in the control group and performed a t-test to assess the statistical significance.

To calculate the specificity and sensitivity of a specific experiment, we compared the YAMTAD system screening results with the experimentally confirmed TCR-pMHC interactions. For the peptide screening, SL9 and 6 variants, 3 F, 3 L, 3H, 3 W, 3Q, and 3 K were enriched in the screening. All the enriched yeasts, except 3 K, can activate the cognate TCR (Fig. 3d). Thus, the specificity of this specific screening was 85.8% (6/7). In addition, 9 mutants among the SL9 variants can activate the 868 TCR, but we only identified 6 variants, so the sensitivity of this specific screening was 66.7% (6/9). For the TCR screening, the specificity and sensitivity were calculated similarly.

### Statistical information

Statistical analyses were performed using GraphPad Prism 8 software. The results are presented as the means ± sd and were considered statistically significant when comparisons between groups were performed using unpaired one- or two-tailed Student’s *t*-test. *Indicates a two-tailed *P* value < 0.05. **Indicates a two-tailed *P* value <0.01. All experiments were performed with at least two independent repeats.

## Supplementary information

Supplementary Information

Supplementary Table 1

supplementary Table 2

supplementary Table 3

Supplementary Table 4

Supplementary Table 5

Supplementary Table 6

## Data Availability

All the data used in this study have been deposited in the National Center for Biotechnology Information (NCBI) BioProject. BioProject Accession number: PRJNA755486. BioSample Accession numbers: SAMN20924278, SAMN20924297, SAMN20924298, SAMN20924299, SAMN20924300, SAMN20924301, SAMN20924302, SAMN20924303.
