# Supplementary material for: Rapid screening of TCR-pMHC interactions by the YAMTAD system
Source: Cell Discov. 2022 Apr 5;8:30. doi: 10.1038/s41421-022-00386-2 (PMC8979966; doi:10.1038/s41421-022-00386-2)
Supplement: Supplementary file 1 — Supplementary Information [file 41421_2022_386_MOESM1_ESM.pdf]

Supplementary Figures.

Fig. S1

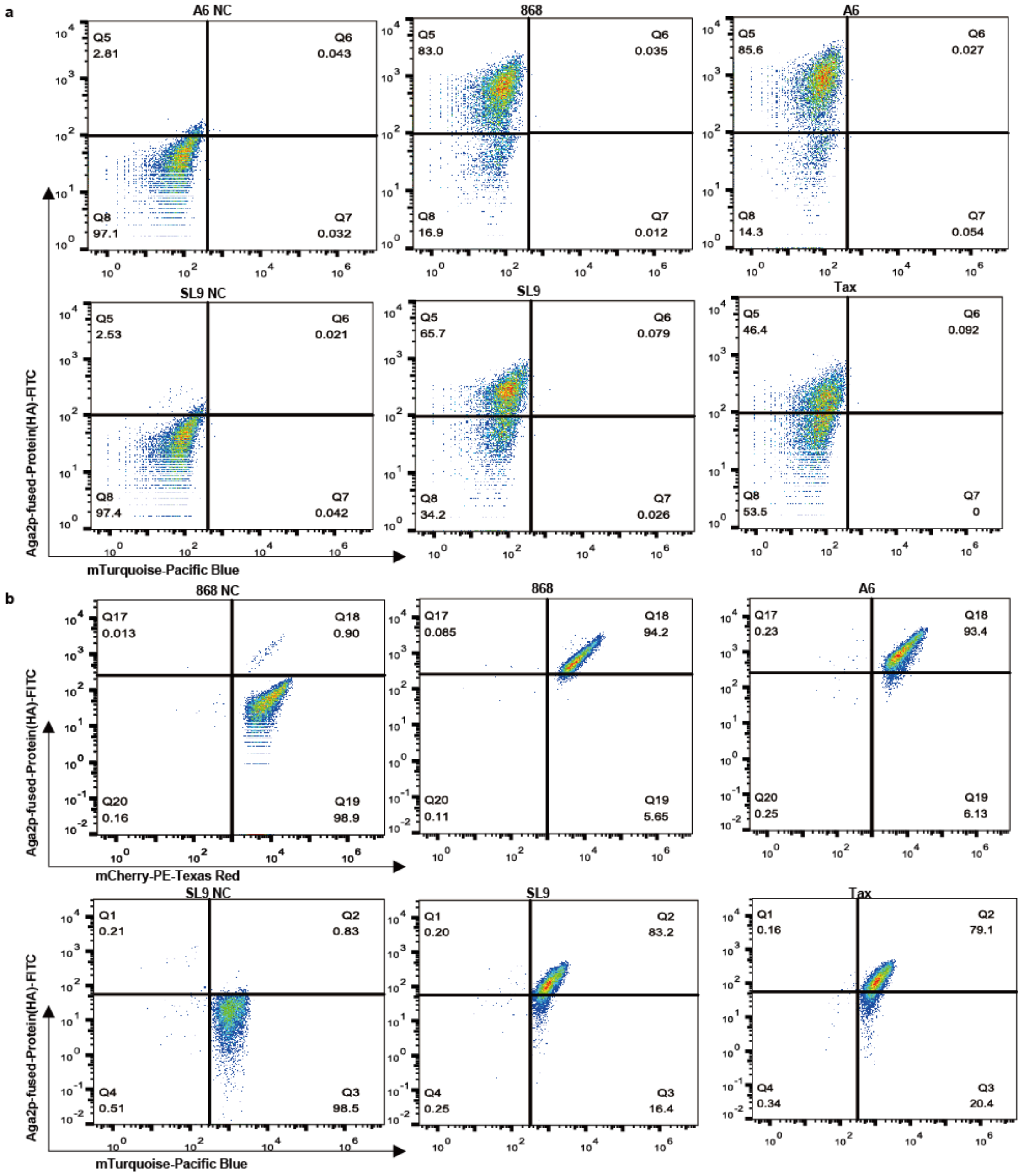

Supplementary Fig. S1 Flow cytometry plot for detecting the display efficiency of TCR and pMHC

a,b Flow cytometry plot of the 868/A6 TCR and SL9-HLA-A*02:01/TAX-HLA-A*02:01 expressed in plasmid form at 22 h (a) or in integrated form at 4 h (b). The negative control (NC) for each group stands for the unlabeled yeast cells. HA was tethered to the Aga2p-fused-TCR/pMHC, which can reflect the display efficiency of TCR/pMHC. Representative data for one out of three biological replicates are shown.

Fig. S2

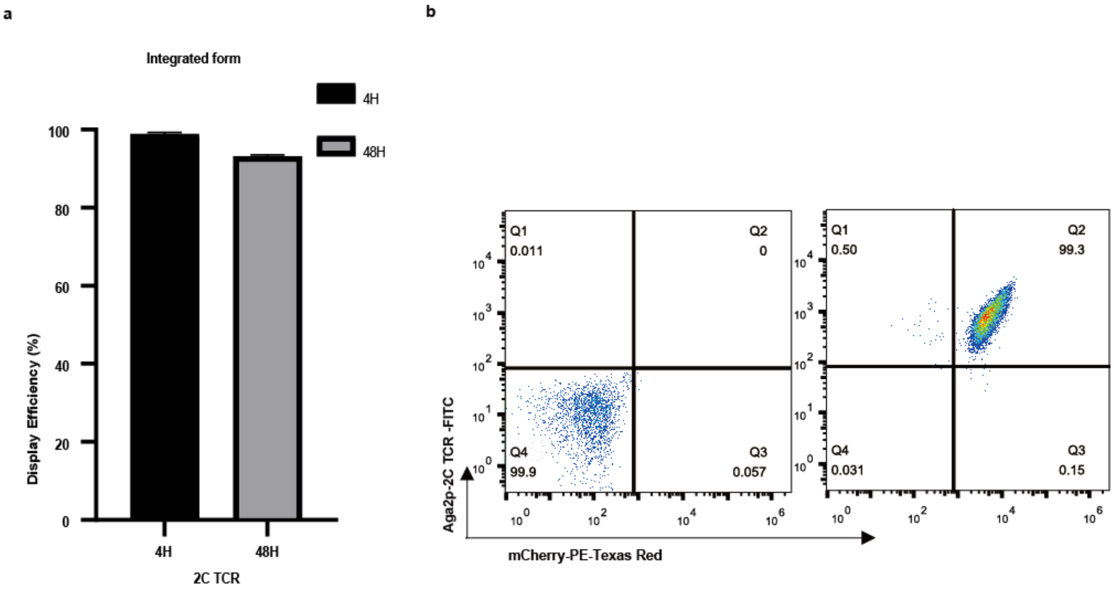

Supplementary Fig. S2 2C TCR can be constitutively expressed to achieve a near-perfect display efficiency with function.

a The display efficiency (mean±s.d.) for 2C TCRs expressed in the integrated form at 4 h or 48 h after culturing in YPD medium. Three biological replicates were measured. **b** Flow cytometry plot for the functional detection of the displayed 2C TCR. The anti-2C TCR-FITC antibody was used to stain the properly folded 2C TCR. Representative data from one out of three biological replicates are shown.

Fig. S3

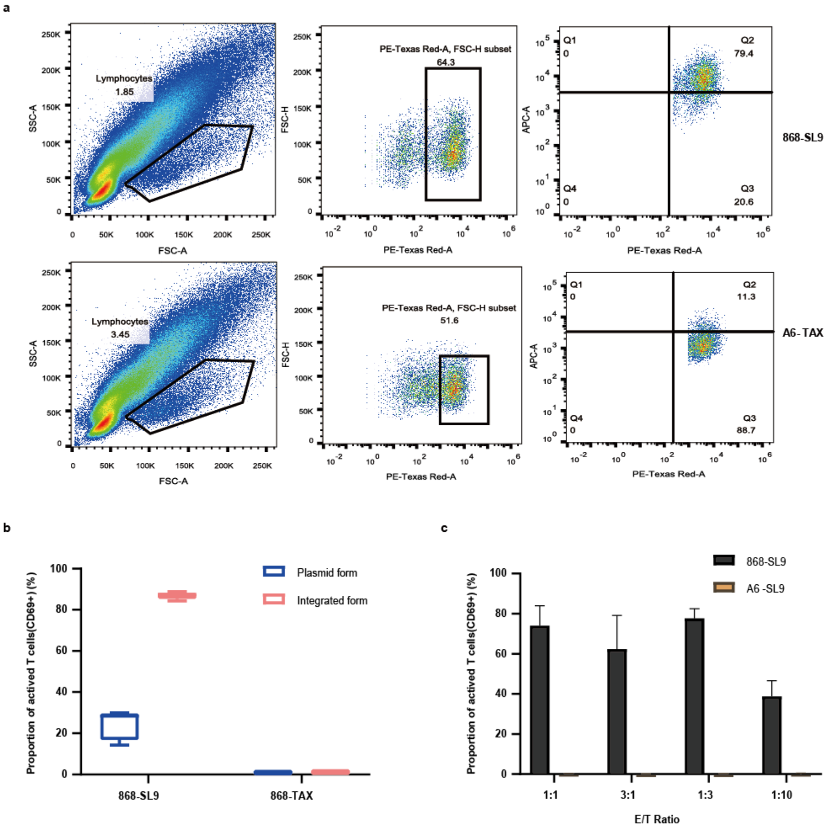

Supplementary Fig. S3 The function of pMHC was confirmed by the yeast T cell co-culture system.

a Flow cytometry gating strategy for calculating the proportion of activated T cells (CD69+) in the yeast T cell co-culture system. The cells were first gated on FSCs/SSCs, followed by gating on the T cell region expressing mCherry. Then, the cells were further gated for analysis of the percentage of the APC+ population (T cells expressing CD69 cells).

b Compared with yeast expressing pMHC in plasmid form, yeast expressing pMHC in integrated form can activate a higher proportion of T cells (CD69+) (mean±s.d.). Each group was set with 2 technical repeats and independently duplicated 3 times. The mean of 6 duplicates is shown.

c Comparison of the proportion of activated T cells (CD69+) (mean±s.d.) in the yeast T cell co-culture system with different E (T cell): T (pMHC-displaying yeast cell) ratios. Each experiment was performed with 2 technical repeats and was independently duplicated 3 times. The mean of 6 duplicates is shown.

Fig. S4

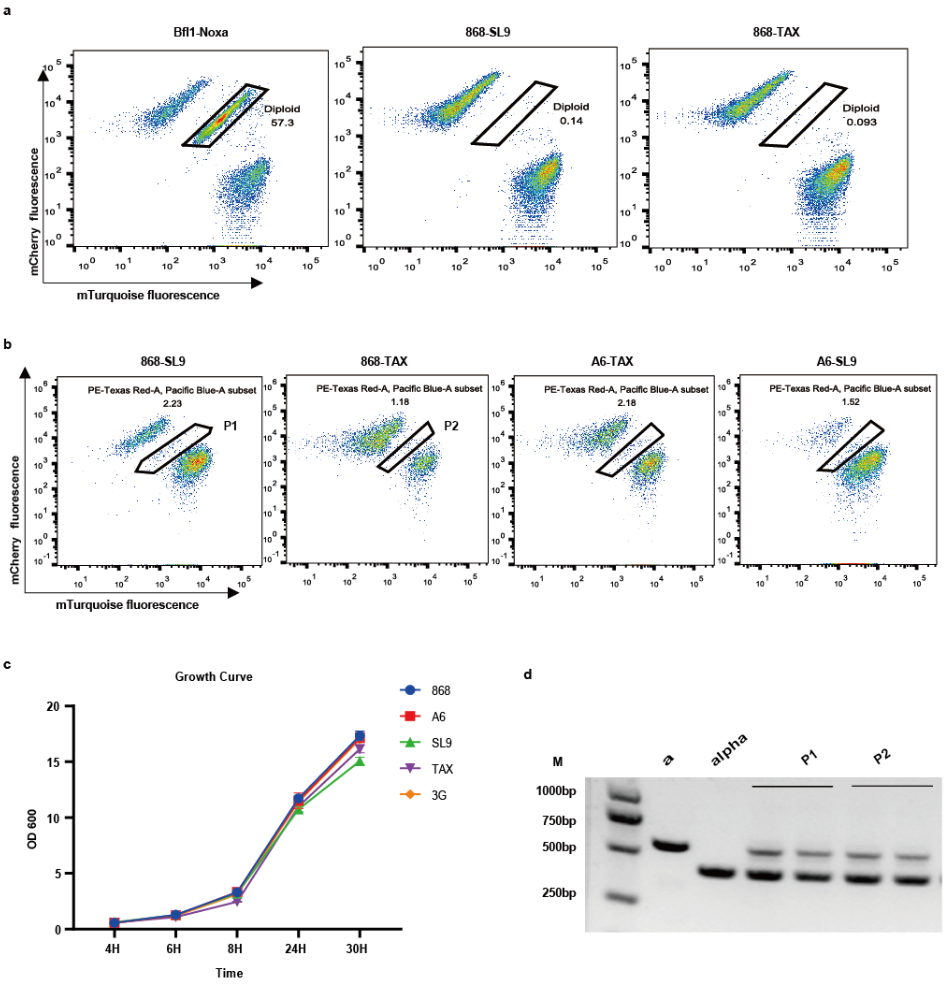

Supplementary Fig. S4 The YAMTAD system can be used for pairwise TCR–pMHC interaction detection

a,b FACS plot for detecting the mating efficiency of different TCR-pMHC pairs using the condition used previously (a) or the condition optimized in our study (b). MATalpha yeast can be detected by mTurquoise fluorescence. MATa yeast can be detected by mCherry fluorescence. The oblique triangle shows the gate for counting diploids (double fluorescence). Representative data for one out of seven biological replicates. Although the gate for the diploid and the a/alpha ratio was highly variable in the flow cytometry analysis, the percentage of diploid was significantly higher in the interacting TCR-pMHC group, as calculated by 7 independent repeats.

c The growth curve (mean±s.d.) of different yeast strains cultured in YPD medium.

d PCR confirmation of the diploid region using the unique primer for MATa and MATalpha.

Fig. S5

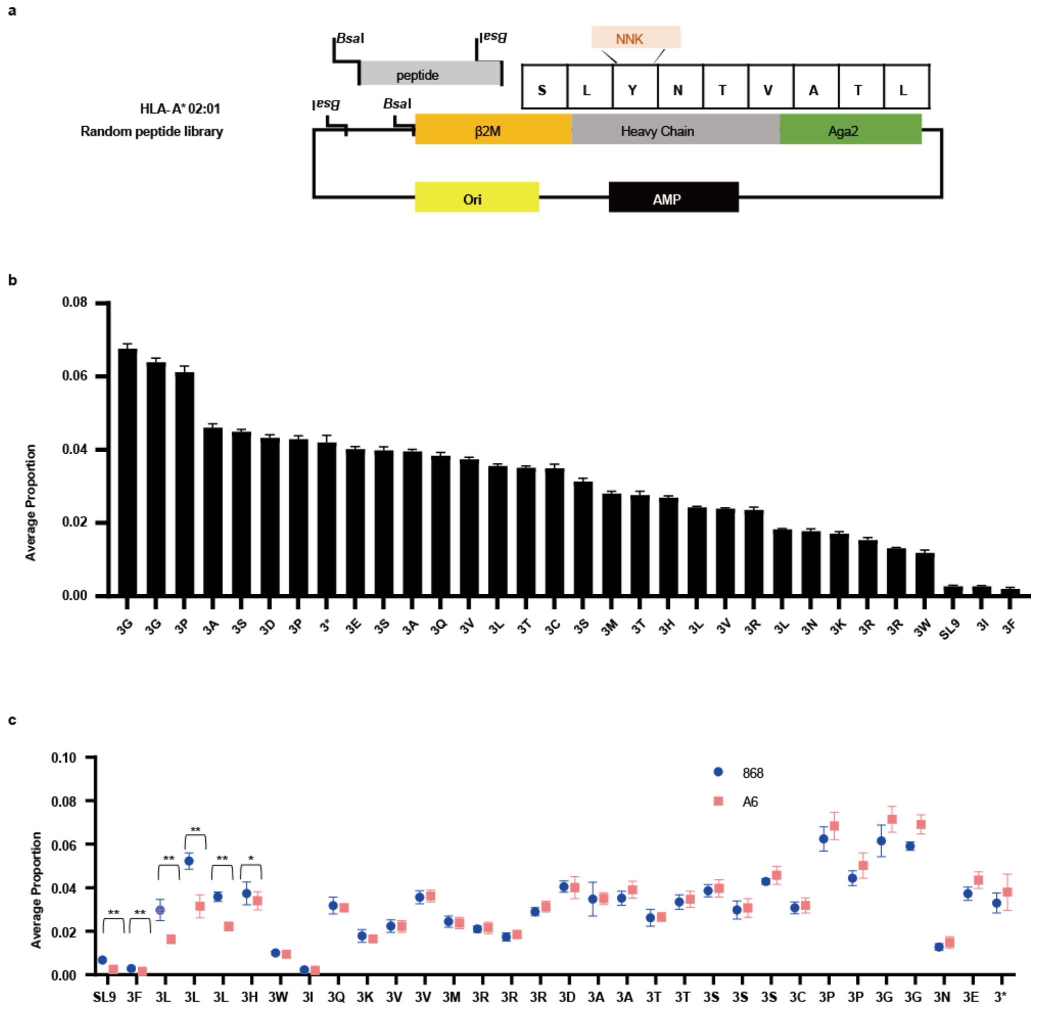

Supplementary Fig. S5 The YAMTAD system can be used for random peptide library screening

a Schematic diagram of the construction strategy for the random library for the SL9 third site mutants.

b Proportions (mean±s.d.) of all mutants in the random library. The mutants were not equally distributed because the primer was random and the library size was not sufficiently large.

c Proportions (mean±s.d.) of all SL9 mutants after YAMTAD screening in the random library. Each experiment was performed with 4 technical repeats and was independently duplicated 3 times. The mean of 12 duplicates is shown.

Statistical analysis of quantification was performed using the unpaired one-tailed Student's t-test. ** Indicates a P value <0.01. * Indicates a P value <0.05.

Fig. S6

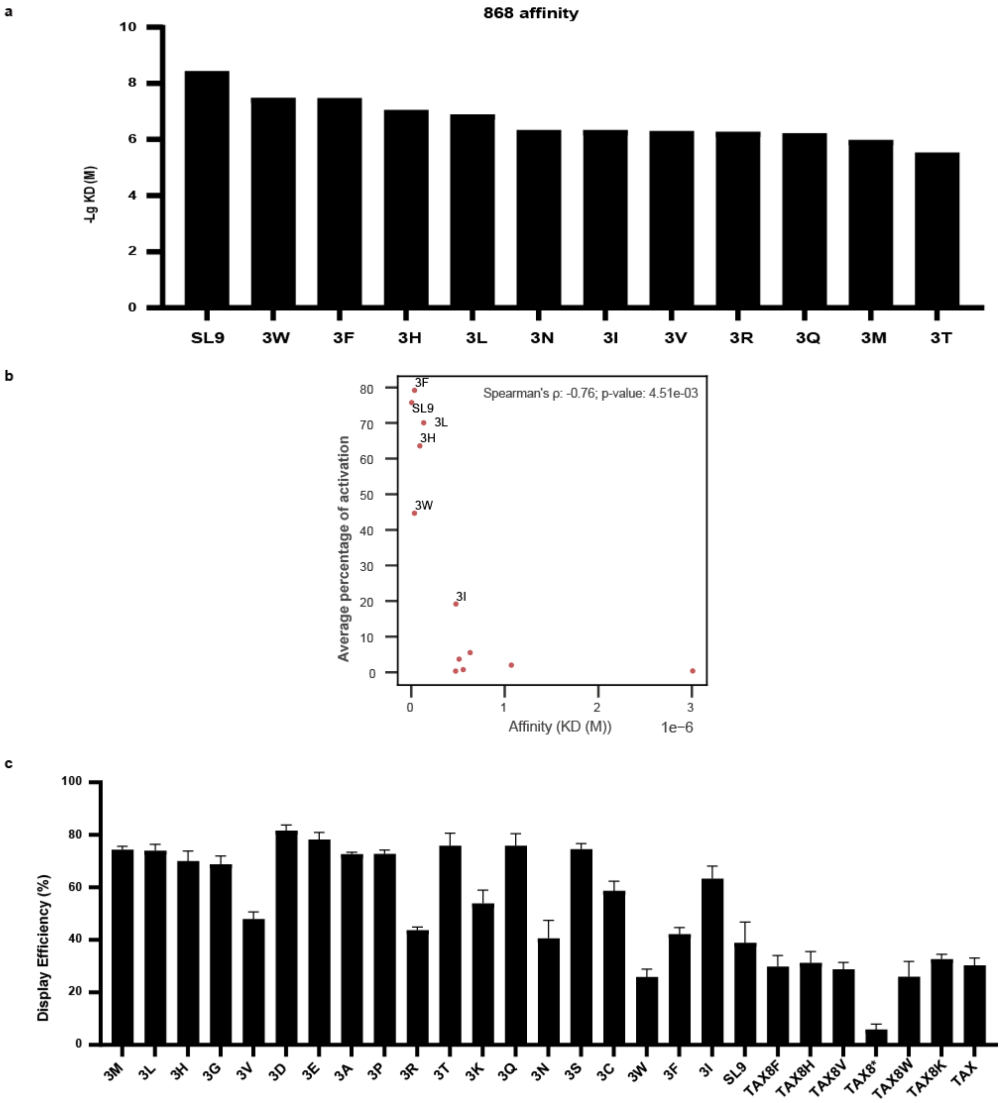

Supplementary Fig. S6 The affinity and display efficiency for all pMHC peptide mutants

a The bs-868Z11-CD3 binding affinity against SL9 peptide SLYNTVATL and peptides from a positional scanning library measured by Andreas Moritz et al[36].

b The correlation between the average percentage of activation and the affinity (KD(M)).

c The display efficiency (mean±s.d.) of all the SL9 mutants was measured after 4 h of culture in YPD medium at OD=0.1.

Fig. S7

a

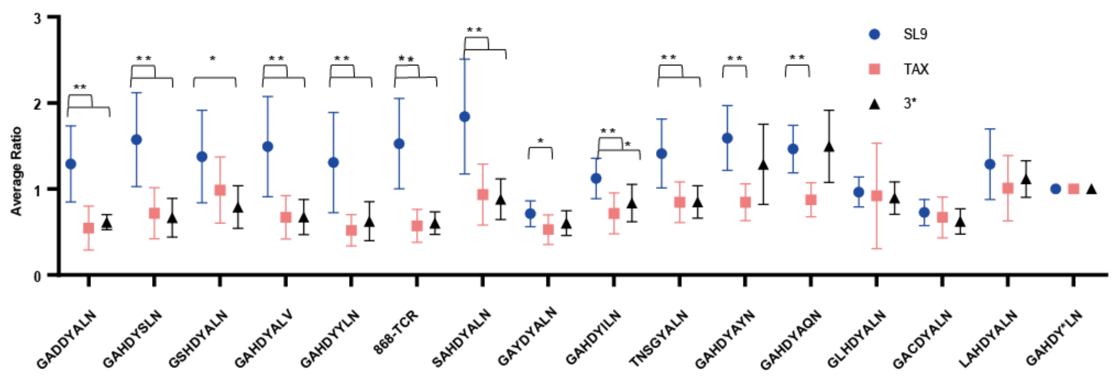

b

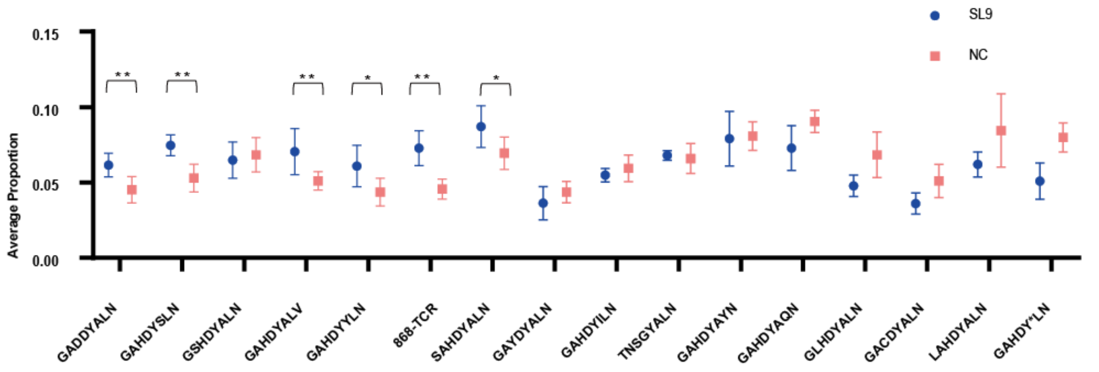

Supplementary Fig. S7 The YAMTAD system can be used for cognate TCR screening given a peptide

a Ratios (mean±s.d.) of the 868 TCR mutants to the GAHDY*LN TCR in diploid yeasts induced in the YAMTAD screening. Each experiment was performed with 4 technical repeats and was independently duplicated 2 times. The mean of 8 duplicates is shown.

b Proportions (mean±s.d.) of all TCR mutants after YAMTAD screening. The proportion of all TCR mutants in the NC group was calculated by averaging the proportions in the TAX and 3* control groups. Each experiment was performed with 4 technical repeats and was independently duplicated 2 times. The mean of 8 duplicates is shown.

a,b Statistical analysis of quantification was performed using the unpaired two-tailed Student's t-test. ** Indicates a two-tailed P value <0.01. * Indicates a two-tailed P value <0.05.

Fig. S8

a

868-barcode 1	CCGAATTGAGATGCA
868-barcode 2	CAAAGATCCTTGGCC
A6-barcode 1	TCCACCCTGCTACGC
A6-barcode 2	CGCCAGGTGTCCGCC
2C-barcode 1	TTCGTTTACCGGTCT
2C-barcode 2	CATAGCACCGCTCGC
SL9-barcode	TTGCTCTGCTAGAAG
TAX-barcode	AGGCAATCACCCAAC
TAX 8* -barcode	ACCGACTGGGCCCGG
3L-barcode	TGTTAGACCCCGCCC
3*-barcode	GGTTAGAGAGATCCC

b

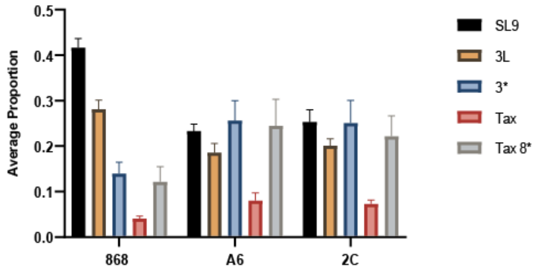

Supplementary Fig. S8 The YAMTAD system can be used for library-on-library screening

a Sequence of the unique barcode used for distinguishing the TCR and pMHC mutants.

b Proportions (mean±s.d.) of each mutant simultaneously appearing with 868/A6/2C after YAMTAD library-on-library screening. Each experiment was performed with 4 technical repeats and was independently duplicated 3 times. The mean of 12 duplicates is shown.

Supplementary Tables.

Supplementary Table S1	Random peptide library screening
Supplementary Table S2	Cognate TCR mutants screening
Supplementary Table S3	Library-on-library screening
Supplementary Table S4	Plasmids used in this study
Supplementary Table S5	Yeast strains used in this study
Supplementary Table S6	Primers used in this study
